# Energy and protein intake in medical and geriatric inpatients with MEDPass versus conventional administration of oral nutritional supplements: study protocol for the randomized controlled MEDPass Trial

**DOI:** 10.1186/s13063-021-05145-4

**Published:** 2021-03-16

**Authors:** Silvia Kurmann, Emilie Reber, Maria F. Vasiloglou, Philipp Schuetz, Andreas W. Schoenenberger, Katja Uhlmann, Anna-Barbara Sterchi, Zeno Stanga

**Affiliations:** 1 Health Division, Department of Nutrition and Dietetics, Research and Development, Bern University of Applied Sciences, Murtenstrasse 10, CH-3010 Bern, Switzerland; 2 Department of Diabetes, Endocrinology, Nutritional Medicine and Metabolism, Inselspital, Bern University Hospital, and University of Bern, Freiburgstrasse 15, CH-3010 Bern, Switzerland; 3 AI in Health and Nutrition Laboratory, ARTORG Center for Biomedical Engineering Research, University of Bern, Murtenstrasse 50, CH-3008 Bern, Switzerland; 4 Medical University Department, Clinic for Endocrinology, Diabetes and Metabolism, Division of General Internal and Emergency Medicine, Kantonsspital Aarau and Medical Faculty of the University of Basel, Tellstrasse H7, CH-5001 Aarau, Switzerland; 5 Department of Geriatrics, Inselspital, Bern University Hospital, and University of Bern, Tiefenaustrasse 112, CH-3004 Bern, Switzerland

**Keywords:** Oral nutritional supplements, Energy intake, Protein intake, Malnutrition, MEDPass, Nutrition as medication, Medication rounds

## Abstract

**Background:**

Disease-related malnutrition is highly prevalent in hospitalized medical and geriatric inpatients. It is associated with negative outcomes such as muscle wasting, decline of functional status, and increased morbidity and mortality. Oral nutritional supplements (ONS) are frequently used in nutritional therapy to increase intake. However, compliance to ONS is often limited and maybe improved by prescribing ONS in small portions timed with the medication (MEDPass). However, it is unknown whether the MEDPass administration enhances patients’ total energy and protein intake.

**Methods:**

The MEDPass Trial is a randomized, controlled, open-label superiority trial. Patients in the MEDPass group receive 50 ml of ONS four times per day, distributed with the medication rounds. Patients in the control group receive ONS between meals. The primary outcome is average daily energy intake (% of calculated daily requirement). For our power analysis, we assumed that administration of ONS in the MEDPass administration mode increases energy intake by at least 10% (i.e., by 200 kcal for an average energy requirement of 2200 kcal/day). Thus, with the inclusion of 200 patients, this trial has 80% power to demonstrate that intervention group patients have an average intake of 2200 kcal/day (SD 500 kcal) versus 2000 kcal/day (SD 500 kcal) in control group patients. Energy and protein intakes from ONS and all food consumed are monitored continuously throughout the hospital stay and are statistically compared to the patient’s requirements. Secondary outcomes include average daily protein intake (% of calculated daily requirement), average intake of ONS/day, the course of body weight, handgrip strength, appetite, and nausea. Furthermore, hospital length of stay and 30-day mortality are assessed. The primary statistical analysis will be performed as an intention-to-treat analysis adjusted for the stratification factors used in randomization.

**Discussion:**

To our knowledge, this is the first randomized controlled trial assessing total energy and protein intake for the entire hospitalization period in patients receiving MEDPass versus conventional ONS administration. Thus, the MEDPass Trial will fill a gap and answer this relevant clinical question.

**Trial registration:**

ClinicalTrials.gov NCT03761680. Registered on 3 December 2018. Kofam.ch SNCTP000003191. Registered on 15 October 2018

## Background

Disease-related malnutrition (DRM) in hospitalized patients is a common problem with an estimated prevalence of 20–60% [1–3]. DRM is associated with detrimental clinical and metabolic consequences, such as catabolism, muscle wasting, impaired muscle function, and mobility [4, 5]. DRM is also associated with higher mortality and morbidity rates and increased hospital length of stay (LOS) [3, 4, 6]. These outcomes not only affect quality of care, but also pose a substantial economic burden [3].

In inpatients at nutritional risk, an adequate and early nutritional therapy has been shown to be effective, significantly reducing severe complications and 30-day mortality [6, 7]. Oral nutritional supplements (ONS) are one of the most common treatments for DRM, and ONS are designed for this specific medical purpose [8, 9]. The use of ONS has proven efficient and cost-effective [10, 11]. Treatment with ONS may reduce mortality and complications, attenuate or prevent muscle loss, and improve nutritional status and function [10, 12, 13]. The European Society of Clinical Nutrition and Metabolism (ESPEN) recommends the use of ONS for medical and geriatric patients at nutritional risk [10, 14]. These patients often live with chronic illness, which is associated with appetite and body weight loss. Thus, the risk of DRM is increased [10].

Compliance with ONS is often described as suboptimal. This poses a barrier to adequate oral nutrition therapy [14, 15]. The ONS administration mode may be one of the keys to improving compliance. There are no standards on how to administer ONS in terms of timing and dose throughout the day, which leads to individual approaches. In the hospital setting, ONS are conventionally served by nurses, nursing aids, or catering personnel between main meals. The MEDPass administration mode offers a different approach, in which ONS are distributed on medication rounds three or four times per day in unusually small portions (50–120 ml) [16–22]. Preliminary trials suggest that the MEDPass administration mode improves compliance and cost effectiveness [17, 19, 22, 23].

However, since ONS are dense in energy and protein, they may have a negative impact on appetite [24]. Consequently, enhancing compliance with ONS may not automatically lead to improved nutritional intake throughout the day because conventional food intake may decline. Therefore, this trial will study total energy and protein intake throughout the day as primary outcome measures when ONS are administered in the MEDPass mode versus the conventional administration mode. To our knowledge, there has never been a prospective trial in which total energy and protein intake was studied consistently and systematically. Therefore, the aim of this trial is to fill this clinically relevant gap of knowledge.

## Methods

### Study design

The MEDPass Trial is a randomized, controlled, clinical trial (RCT) conducted with parallel groups. Blinding of patients is not possible because of the evident differences in ONS administration times. Therefore, it is conducted as an open-label RCT. The MEDPass Trial is a superiority trial. The allocation ratio of intervention group and control group is 1:1.

### Study population

The MEDPass Trial includes medical as well as geriatric inpatients at the Tiefenau facility of the University Hospital of Bern. It includes inpatients of the Department of Geriatrics and the Department of General Internal Medicine who meet the eligibility criteria listed in Table 1.

**Table 1:** Inclusion and exclusion criteria

**Inclusion criteria**	• Nutritional Risk Screening (NRS 2002) total score ≥ 3 points according to routine screening at admission within 72 h [25] • Expected hospital LOS ≥ 3 days after screening (as estimated by the treating physician) • Patient qualifies for ONS and approves prescription • Age > 18 years • Willingness and ability to provide informed consent
**Exclusion criteria**	• Initially admitted to critical care unit • Immediate post-operative phase (< 7 days post-surgery) • Dysphagia with the inability to swallow liquids • Admitted with or scheduled for supplemental or total enteral nutrition and/or parenteral nutrition • Mini Mental State examination < 16 points • Hospitalization due to anorexia nervosa, acute pancreatitis, or acute liver failure • Patients with cystic fibrosis, short bowel syndrome, or after gastric bypass surgery • Terminal condition (end of life situation) • Poor German language skills (study language)

### Study intervention and comparator

Every patient in the MEDPass group receives 50 ml of ONS four times per day distributed by the registered nurses (RN) on the ward during medication rounds. Three out of the four medication rounds occur approximately 30 min before the main meals’ distribution, and the evening medication round is at 10 pm. Patients are counseled to take the ONS directly at the start of the meal to avoid any possible interaction with medications. The control group regimen was chosen to compare the MEDPass administration mode to usual clinical practice. Usual practice of ONS administration at the study site is unstandardized. This kind of prescription may range from one up to four bottles of ONS per day and is served between the meals or after the evening meal. Patients in the control group receive ONS between meals according to unstandardized prescriptions. The staff was instructed not to make any changes to the procedures followed before the initiation of the trial. This means that the amount of ONS prescribed to control group patients might be higher than those of the intervention group. However, compliance with ONS may be better with the MEDPass-mode. Compliance in the context of the MEDPass Trial is defined as the percentage of prescribed ONS consumed. Furthermore, patients receiving ONS in the MEDPass administration mode may be able to eat more additional food between meals than those receiving ONS between meals. Thus, we hypothesize that MEDPass group patients are more likely to reach their energy and protein targets even if their net prescription of ONS is lower.

### Type of ONS used in the MEDPass trial

All ONS used in this trial are selected according to patients’ nutritional needs and flavor preferences. Patients may choose from a wide range of available products at University Hospital of Bern. All products with an energy density of 1.5 kcal/ml and 2 kcal/ml are included in this trial. Overall, eight different ONS with this energy density are in stock. The amount of protein in these ONS ranges from 4 to 10 g/100 ml. Six of the ONS are nutritionally complete. Two of them do not contain any fat. The ONS are from different providers (Abbott Nutrition, Fresenius Kabi, Nestlé Health Science). If patients require an ONS that is not available in the standard assortment of the University Hospital, it can be ordered if it is obtainable on the Swiss market.

### Ethical aspects

The study is carried out in accordance with the current version of the World Medical Association Declaration of Helsinki protocol [26], ICH-GCP guidelines [27, 28] or ISO 14155 norm [29] and according to the Swiss Federal Act on Research involving Human Beings [30, 31].

### Randomization

Randomization is stratified according to the NRS 2002 total score and the energy content of the ONS (kcal/ml). The NRS 2002 total score is stratified as NRS 3, NRS 4, or NRS 5-7. The energy content of the ONS is stratified as either 1.5 kcal/ml or 2 kcal/ml. Even distribution of these factors between the groups minimizes bias since they may directly influence primary and secondary outcome parameters. Randomization is conducted using the Research Electronic Data Capture (REDCap®) program (Vanderbilt University, Nashville, TN, USA, 2020, version 9.1.15). Registered Dietitians (RD) input the stratification data and randomize electronically within the REDCap® program. The RDs do not have access to the concealed randomization sequence. The randomization list with random numbers for coding was pre-specified by the Clinical Trial Unit (CTU) Bern and electronically integrated into the REDCap® database.

### Outcomes

The primary outcome is the average energy intake/day (kcal, % of calculated daily requirement). Secondary outcomes include average protein intake/day (g, % of calculated daily requirement), average ONS intake/day (ml), the course of handgrip strength (kg), body weight (kg), appetite, and nausea. Furthermore, hospital LOS and 30-day mortality are assessed.

### Study conduct and data collection

**Fig. 1:**
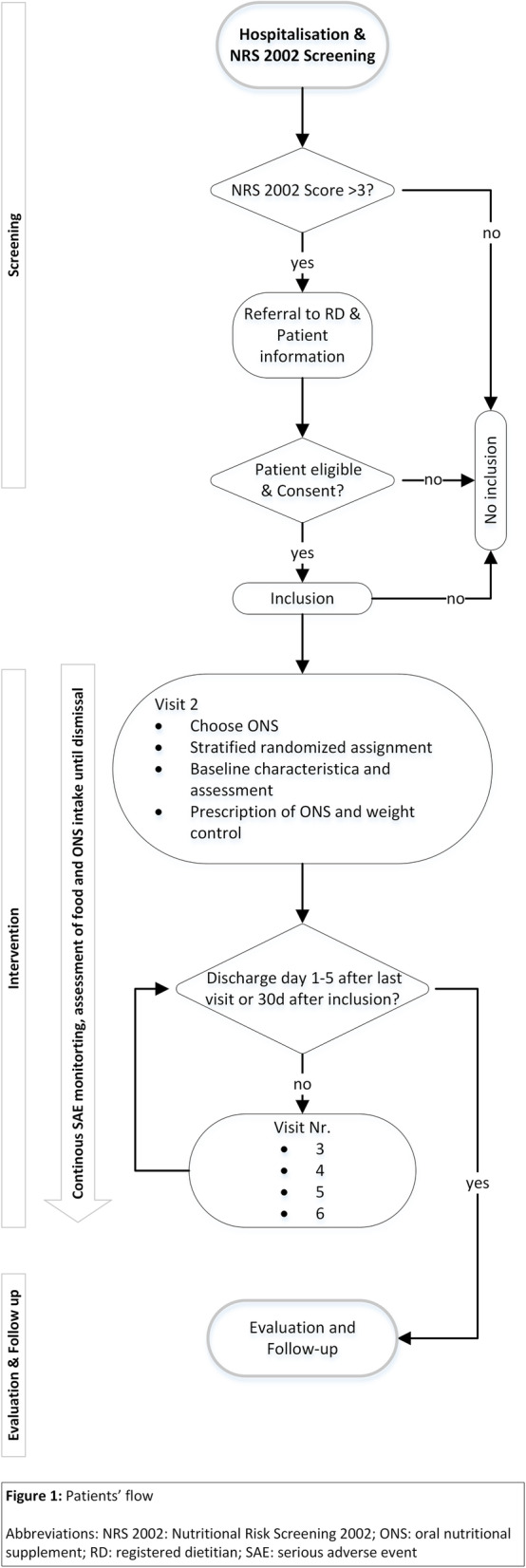
Patients’ flow. NRS 2002, Nutritional Risk Screening 2002; ONS, oral nutritional supplement; RD, registered dietitian; SAE, serious adverse event

Participants are recruited consecutively. Based on routine screening upon admission, patients with an NRS 2002 total score of > 3 are referred to the RDs [25, 32]. The RDs check the eligibility criteria. If there is an indication but no ONS prescribed, the RDs counsel the patient concerning ONS. Once an eligible patient agrees to a prescription of ONS and an appropriate product is selected, the patient is informed by the RD about the possibility of participating in the trial. Upon approval, the RD obtains informed consent and randomizes the patient. The prescription of ONS is carried out by the medical doctors (MD). All involved personnel (MDs, nursing and catering staff) is informed by the RDs of the patient’s participation in the trial. An overview of the patients’ flow is given in Fig. 1 and an overview of the time schedule for participants is provided in Table 2. The trial is only concerned with ONS administration and has no influence on any other aspects of patients’ treatments. ONS may be discontinued if the indication therefore no longer exists.

**Table 2:** Time schedule for participants

Study period	Screening	Intervention period					Evaluation	Follow-up
Visit	1	2	3	4	5	6		
Day	0	1	8	15	22	29	*	**
NRS 2002 total score	x							
NRS 2002 subscore nutritional status	x							
Inclusion and exclusion criteria	x							
Information, informed consent	x							
Assignment to study group		x						
Baseline patient characteristics		x						
Energy intake/day (kcal)**		Continuous assessment						
Protein intake/day (g)**								
ONS intake/day (ml)**								
Serious adverse events (SAE)**								
Handgrip strength (kg)		x	x***	x***	x***	x***		
Body weight (kg)		x	x***	x***	x***	x***		
Body height (cm)		x						
BMI (kg/m^2^)		x						
Appetite (VAS)		x	x***	x***	x***	x***		
Nausea (VAS)		x	x***	x***	x***	x***		
Procedural variables							x	
Hospital LOS (d)							x	
30-day mortality (y/n)								x

*After patient dismissal or after 30 days of inclusion

**For 30 days upon inclusion

***Assessment at day 1, day 8, day 15, day 22, and day 29

Visits 2–6: study visits may be conducted earlier or later by a maximum of 2 days

The criteria for ONS discontinuation are:

- ONS is not indicated anymore due to increased food intake
- Occurrence of a serious adverse event (SAE) that necessitates a halt to the prescription

The reasons for discontinuation are documented. Furthermore, withdrawal from the trial is possible at any time at the patients’ request.

All data is inputted manually by the RDs into REDCap. REDCap is a program that provides the highest standard of data protection and an internal back-up system is in place to prevent data loss. The REDCap study database with all archive tables will be securely stored by CTU Bern for at least 15 years. The sponsor also keeps the Trial Master File (TMF) and interim/final reports for at least 10 years. All extracted data files are exclusively stored on password-protected personal computers. Source data is collected mostly from the electronic health record (EHR). This includes the NRS 2002, in- and exclusion criteria, patient characteristics, ONS intake, body weight, LOS, and safety outcomes. Meal cards, forms for collecting data on food intake between the meals and protein and energy intake calculations, are printed out and input manually into REDCap by the RDs. All trial-related paper documents are locked in a specific cabinet in the RD’s office during the whole study period. Afterwards, this material will be stored safely for 10 years in the locked archive of the Department of Endocrinology, Diabetes, Nutritional Medicine, and Metabolism at Bern University Hospital.

All participating staff have received formal training for all their tasks, and training materials are available on site on the intranet platform. Furthermore, training is provided continuously due to staff turnover. Regular exchange with appointed members of nursing and medical staff is implemented to monitor adherence to the protocol. The MEDPass Trial is managed and overseen by the study steering committee, the dietetic committee, and the data management committee. The study steering committee (authors SK, ER, PS, AWS, ABS, and ZS) is responsible throughout for main decisions on conduct including risk management, reports to the Competent Ethics Committee (CEC), and results dissemination. The study steering committee may bring forward suggestions for protocol amendments. If approved by the committee, they will be reported to the CEC. Substantial amendments are only implemented after CEC approval.

Under emergency circumstances, deviations from the protocol to protect the rights, safety, and wellbeing of human subjects may proceed without prior approval of the sponsor, the study steering committee, and the CEC. Such deviations shall be documented and reported to the sponsor, the steering committee, and the CEC as soon as possible.

All non-substantial amendments are communicated to the CEC within the Annual Safety Report (ASR). The trial registries will be updated within 30 days. Trial participants will be contacted via written communication and will be given the possibility to withdraw their approval.

The dietetic committee (authors SK, ABS and the team of RDs on site) oversee the implementation of recruitment, data collection, and the ongoing training sessions. The data management committee (authors SK, ER, MV, PS, AWS, and ZS) monitor data quality and are responsible for the statistical plan. During the recruitment period, an independent employee from Bern University of Applied Sciences will act as monitor, conducting at least one monitoring visit of the trial on site. The frequency and duration of the visits are dependent on the rate of subject enrollment, the quality of the study documents and the findings. During the visit(s), the monitor will check whether the trial is being carried out according to the study protocol and whether the subjects’ safety and rights are being protected. Furthermore, the monitor will review the collected trial data and verify source documents. The sponsor and the principal investigator on site allow the monitor direct access to all relevant documents during visits. They also agree to allocate their time and the time of their staff to the monitor to discuss findings and any other relevant issues.

In case of discrepancies, the monitor will make recommendations and schedule visits at shorter intervals. For each contact, a report is completed and signed by the monitor and the sponsor.

As category A trials are exempt from providing insurance, there is no specific insurance policy in place. However, general liability insurance is provided by Inselspital, Bern University Hospital.

### Measurements

#### Energy, protein, and ONS intake

All parameters are assessed continuously until discharge or for a maximum of 30 days (Table 2). The RDs calculate daily energy and protein requirements according to relevant current clinical nutrition guidelines that apply to the patients in the MEDPass Trial [10, 14, 33]. The formula per patient is chosen according to age and body mass index (BMI). As some patients may suffer from progressive renal failure, the Clinical Practice Guideline of the National Kidney Foundation is used to calculate these patients’ protein needs [34]. The formulas for daily energy requirements as related to the primary outcome are listed in Table 3.

**Table 3:** Calculation of daily energy requirements

Formulas for daily energy requirements		
	BMI < 18.5 kg/m^2^	BMI > 18.5 kg/m^2^
< 65 years	Actual body weight × 30 kcal [10]	Actual body weight × 27 kcal [10]
> 65 years	Actual body weight × 32 kcal [14]	Actual body weight × 30 kcal [14, 33]

Recommended protein intake is calculated according to the guidelines for geriatric and medical patients using the formula 1 g/kg body weight/day [10, 14, 33]. However, patients with chronic kidney disease and estimated glomerular filtration rate (eGFR) < 30 mL/min/1.73m^2^, who are not on dialysis, are an exception. In this case, the actual body weight is multiplied by 0.8 g/kg body weight/day [34].

Detailed data concerning food intake is assessed after every meal. Each component of every meal is evaluated separately by the personnel collecting the tray after the meal. More specifically, the consumed percentage (100%, 75%, 50%, 25%, or 0%) of any delivered food component is evaluated and the value is noted accordingly on the meal card. Furthermore, after each meal, patients are interviewed on food intake between the main meals. To keep track of intake in addition to meals, a separate document is used. It is distributed and collected during morning meal rounds. All food intake documentation is temporarily stored at ward level and collected daily by the RDs from Monday to Friday. For the weekends, it is collected on Mondays. If data points are missing, the RDs interview the patient directly to fill the respective data gaps. All meals at the facility are prepared according to recipes of the Bern University Hospital database. Energy and protein intakes are primarily calculated via the electronic menu system LogiMen® (Kretschmer-Keller Leonberg, Germany, 2016, version 5.4). The system contains energy and protein content data for all hospital meals and snacks. This method ensures high calculation accuracy as the recipes are integrated in the system. Energy and protein intake from food items that are not registered in the LogiMen® system are calculated by the RDs using nut.s nutritional software® (dato Denkwerkzeuge, Vienna, Austria, 2008, version 1.32.74). All meal cards and calculations are sent to one of the co-investigators, who is blinded to patient allocation, for recalculation.

The amount of ONS consumed is monitored daily by the RNs by measuring the amount consumed with a measuring cup. In the trial setting, ONS is prescribed and listed in the medication chart in the EHR. All medications are listed with the amount prescribed. The amount consumed is documented on a standardized basis for all medications. The consumed amount is documented in the EHR with an accuracy of ± 5 ml. From this assessment, the RDs calculate energy and protein intake from ONS.

#### Handgrip strength

Handgrip strength is assessed by the RDs on study visits 2–6 (Table 2) until discharge or for a maximum of 30 days (Table 2). Handgrip strength is evaluated using the JAMAR® Hydraulic Hand Dynamometer (Patterson Medical, Warrenville, IL, USA) with an accuracy of ± 0.5 kg. The measurement is always conducted according to the same procedure and on the same hand. If possible, the measurement is performed on the dominant hand. To ensure data validity, the measurement is taken three times with a break of at least 30 s between measurements and the highest value is noted [35].

#### Body weight

Body weight is monitored as part of standard clinical practice by the nursing staff with an accuracy of ± 0.1 kg. Immobile patients are weighed on the seca® wheelchair scale (Vogel & Halke, Germany, model 665) and mobile patients on the sitting scale seca® (Vogel & Halke, Germany, model 959). Body weight measurements are prescribed by the MDs according to the study schedule (Table 2) directly after patient enrolment in the trial. RDs evaluate if body weight measurements are accurate according to the patient’s clinical status and medications. RDs may contact the MDs to discuss the validity in case of uncertainty.

#### Appetite and nausea

The course of appetite and nausea are recorded by the RDs during weekly study visits (Table 2). The level of appetite and nausea on the current day are the focus of the assessment. The assessment is carried out by asking the patient to scale the level of appetite/nausea using a visual analog scale (VAS). The scale visible to the patient only has smileys. The scale on the backside of the smiley scale ranges from 0 to 10 cm so that the subjective measurement can be objectified with an accuracy of ± 0.1 cm.

#### Hospital LOS and 30-day mortality

One of the co-investigators evaluates hospital LOS and 30-day mortality after the participants are released from the hospital or after 30 days of trial inclusion respectively (Table 2). Hospital LOS is assessed from the EHR as number of days: the calculation excludes the day of hospital discharge [36]. Hospital LOS is only assessed in medical inpatients since the geriatric inpatients have a prefixed duration of stay. Assessment of 30-day mortality is done by phone call to the patients’ home or the institution where the patient was referred to. If the patient is still hospitalized, the information is available in the EHR.

#### Baseline patient characteristics and procedural variables

The following baseline patient characteristics are recorded by RDs during visit 1 (Table 2):

- ✓ Disease categories for main diagnosis (gastrointestinal disease, infectious disease, cardiovascular disease, neurological disease, oncologic disease, other diseases)
- ✓ Department in which the patient is hospitalized
- ✓ Gender (male, female)
- ✓ Age (years)
- ✓ Energy content per ml ONS (1.5 kcal/ml or 2 kcal/ml)
- ✓ NRS 2002 total score
- ✓ NRS 2002 subscore for impaired nutritional status
- ✓ Body weight (kg)
- ✓ Body height (cm)
- ✓ BMI (kg/m^2^)

The following procedural variables are recorded after discharge by one of the co-investigators:

- ✓ Number of days from trial inclusion to hospital discharge
- ✓ Number of days with prescribed ONS
- ✓ Number of days with energy and protein intake monitoring
- ✓ Compliance versus non-compliance with the study protocol
- ✓ Number of meals at which patients were nil per os during energy and protein intake assessment (meals at which patients are not allowed to eat for medical reasons)
- ✓ Number of meals at which food intake could not be assessed
- ✓ Involvement of RDs in the treatment of the patients

#### Safety outcomes

As defined by Good Clinical Practice (GCP) [27], SAEs are monitored and investigated continuously. Potential SAEs in this trial are pneumonia caused by ONS aspiration and death associated with pneumonia caused by ONS intake in the MEDPass-mode.

### Statistical analysis

#### Hypothesis

##### Working hypothesis

We hypothesize that patients in the intervention group (MEDPass mode) consume more energy as compared to patients in the control group.

##### Null hypothesis (Ho)

There is no difference in energy intake between the groups.

#### Sample size calculation

Based on our clinical experience, we assumed for our power analysis that administration of ONS in the MEDPass mode increases energy intake (primary endpoint) by at least 10% (i.e., by 200 kcal from an average of 2000 kcal/day). Furthermore, we assumed that an average patient weighs 75 kg and therefore has an approximate energy requirement of 2200 kcal. A final assumption is that in current clinical practice they may only receive 2000 kcal/day. Therefore, to demonstrate that intervention group patients have an increased average daily energy intake of 200 kcal/day (i.e., from 2000 (SD 500 kcal) to 2200 (SD500 kcal)), we need to include 200 patients to achieve 80% power (alpha error of 0.05). The power calculation used the sampsi command in STATA® (Stata Corp, USA, 2017, version 15.3). Importantly, this trial is not powered to investigate whether the difference in energy intake of 200 kcal/day makes a significant difference to clinical outcomes. However, a similar increase in energy intake has previously shown to decrease the risk of adverse outcome and mortality [7].

#### Primary analysis

The baseline patient characteristics will be summarized for both groups. The primary analysis will be performed as an intention-to-treat (ITT) analysis including all randomized patients regardless of protocol adherence. In a further step, we will also do a per-protocol analysis excluding patients with major protocol violations as outlined below.

For the primary outcome, we will test whether MEDPass administration is superior to usual care and compare the average amount of daily energy intake using a Student’s *t* test or chi-square test. We will also fit linear regression models adjusted for initial NRS 2002 score and average amount of energy in the ONS, reported adjusted differences (coefficients), and corresponding 95% confidence intervals (CIs). Total energy, protein, and ONS intake throughout the study period will be divided by days of assessment per patient. Linear regression analysis adjusted for stratification factors will also be used for the evaluation of average daily protein intake as compared to the individual requirement (%) throughout the hospitalization, average intake of ONS/day (ml) throughout the hospitalization, and hospital LOS (days) between the groups. Logistic regression analysis adjusted for stratification factors will be is used for difference in 30-day mortality (yes/no) between the groups. Repeated measure models will be used for the evaluation of differences in course of handgrip strength throughout the hospitalization, weight changes throughout the hospitalization, and course of appetite and nausea throughout the hospitalization. The procedural variables will be analyzed and presented descriptively. There will not be a statistical safety assessment since there are no safety endpoints in the MEDPass Trial. Safety events will be presented descriptively.

### Secondary analyses

#### Per-protocol analysis

A per-protocol analysis will be conducted excluding patients who were incompliant with the study protocol (ONS-prescription for < 80% of the days from inclusion to discharge), patients that were nil per os for > 10% of their meals during the time of intake monitoring, patients of which > 10% of meals could not be assessed during the time of intake monitoring, patients that did not receive the randomized intervention, and patients violating any eligibility criteria.

The same parameters and tests as in the primary analysis will be assessed and conducted for this population.

#### Subgroup analyses

Subgroup analyses on the ITT patient set will include mean energy and mean protein intake throughout the hospitalization as compared to requirements. Linear regression will be used and adjusted for stratification factors to enable comparison of patients by group of different ONS energy content, different NRS 2002 subscore for nutritional status, different levels of appetite and different levels of nausea on day 1, different levels of handgrip strength at day 1, and different levels of dietetic involvement in patients’ nutritional care.

## Trial status

Recruitment is ongoing since November 22, 2018. As of September 8, 2020, 126 participants have been recruited. Expected completion of recruitment according to current projection is November 2021. The trial is conducted according to the protocol version 1.2 of November 22, 2019.

## Dissemination policy

The plans for the dissemination of the MEDPass Trial results include a publication in an international peer-reviewed journal, a poster presentation at one of the renowned nutrition congresses such as ESPEN or NUTRITION and the implementation of the results in the BSc curriculum of Nutrition and Dietetics at Bern University of Applied Sciences.

## Discussion

The MEDPass Trial studies a broad spectrum of patients at nutritional risk. This approach was chosen deliberately to ensure representability of the results on polymorbid patient populations of general medical and geriatric departments. The MEDPass Trial falls under the category of effectiveness research [37]. Effectiveness research aims at broadly representing the studied patient population and compares interventions to usual clinical practice [37]. Thus, the results of effectiveness research support clinical decision making and may improve the quality, effectiveness, and efficiency of health care [37]. A representative patient population as studied in the MEDPass Trial may more efficiently support decision making for the ONS administration mode.

Although the polymorbid, frail patient population accounts for the majority of healthcare costs, it is the least studied population [38]. Polymorbid patients are most often excluded from RCTs because of the complexity of their health condition [38].

To our knowledge, the MEDPass Trial is the first RCT in which total energy and protein intake of subjects with MEDPass versus conventional ONS administration are studied consistently and systematically throughout their hospitalization. Thus, the MEDPass Trial will answer the clinically relevant question if the MEDPass mode of administration is superior to the conventional administration of ONS concerning total energy intake of patients. Furthermore, it may help clinicians in their decisions on administration mode for clinical practice.

As in any open-label trial, there is potential for performance bias, as patients may be influenced by their trial participation [39]. Compliance with ONS and nutritional therapy may thus be increased [39]. In the MEDPass Trial, blinding of participants is not feasible due to the obviously different times of ONS administration. However, the recalculation of all meals by a fully blinded co-investigator enhances objectivity, promotes data quality, and minimizes bias.

The stratification of randomization is aimed at preventing statistical differences between groups due to ONS energy density. This is a relevant approach since differences between groups would directly affect the primary outcome. Furthermore, the stratification for NRS 2002 score is aimed at reducing differences in nutritional risk between groups. This may reduce bias significantly and therefore all statistics will be adjusted for both stratification factors. The wide selection of ONS available in the institution represents daily clinical practice. There is no influence of the financial sponsor on the selection of ONS in this study.

Our initial calculation and projections predicted the completion of patient recruitment in spring 2020. In the course of the trial, recruiting was prolonged for various reasons, including predominantly organizational barriers to recruitment. Organizational barriers are frequently described as significant for the prolongation of clinical trials [40–42]. In patients with unscheduled admissions, such as the majority of those in our facility’s medical department, recruitment challenges are more evident. LOS in the medical department is usually shorter than on the geriatric wards. This disqualifies some of the medical patients because after screening and referral, they may not stay three more days as needed according to the eligibility criteria. Moreover, medical patients with unscheduled hospitalizations may be too anxious about their treatment, in pain, or too unwell to provide consent [42]. Recruitment on the geriatric wards also presents challenges. Reading and understanding the consent forms may be difficult for some of the patients [41]. Furthermore, distrust and/or fatigue are known barriers in recruiting elderly patients onto clinical trials [41].

To facilitate recruitment, Kadam et al. as well as Huang et al. suggest reducing the complexity and interprofessional dependencies in trial setups [43, 44]. As the management of malnutrition is a highly interprofessional task, possibilities for reduction of complexity are scarce. Retrospectively, one option may have been to task the RDs to conduct the nutritional screening. In that scenario, the whole process from screening to patient information to inclusion would have been in the hands of the RDs. This may have proved more efficient as interprofessional dependencies would be reduced. 86% of clinical trials do not reach their recruitment targets in the projected time [43]. As personal resources of all involved professions in the MEDPass Trial are limited, prolonging the recruitment period to reach the desired power was the obvious course of action.

## Data Availability

The datasets used and/or analyzed during the current study are available from the corresponding author on reasonable request and with permission of all authors.

## Abbreviations

BMI: Body mass index
CEC: Competent Ethics Committee
CTU: Clinical Trial Unit
DRM: Disease-related malnutrition
EHR: Electronic health record
ESPEN: European Society of Clinical Nutrition and Metabolism
GCP: Good Clinical Practice
ITT: Intention-to-treat
LOS: Length of stay
MD: Medical doctor
NRS 2002: Nutritional Risk Screening
ONS: Oral nutritional supplements
PN: Parenteral nutrition
REDCap®: Research Electronic Data Capture
RCT: Randomized controlled trial
RD: Registered dietitian
RN: Registered nurse
SAE: Serious adverse event
TMF: Trial Master File
VAS: Visual analog scale
